# The Role of Nanomaterials and Nanotechnologies in Wastewater Treatment: a Bibliometric Analysis

**DOI:** 10.1186/s11671-018-2649-4

**Published:** 2018-08-10

**Authors:** Meng Jiang, Yun Qi, Huan Liu, Yinguang Chen

**Affiliations:** 1 State Key Laboratory of Pollution Control and Resources Reuse, School of Environmental Science and Engineering, Tongji University, 1239 Siping Road, Shanghai, 200092 China; 2 Faculty of Environmental Science and Engineering, Tianjin University, No. 92, Weijin Rd., Nankai District, Tianjin, 300072 China; 3 China’ Three Gorges Projects Development Co., Ltd, No. 288, Fucheng Avenue, High-tech District, Chengdu, 610041 Sichuan China

**Keywords:** Nanomaterials, Nanotechnologies, Wastewater, Treatment, Bibliometric analysis

## Abstract

Nanomaterials and nanotechnologies (NNs) have been shaping the wastewater treatment process unprecedentedly. Bibliometric methods are regarded as an indispensable light to guide direction in scientific domain. The present study aims to investigate the role of NNs in wastewater treatment with bibliometric techniques based on SCI databases from 1997 to 2016. Results showed that China (962), USA (324) and Iran (140) are the most productive countries. Chinese Academy of Sciences (149), Tongji University (49), and Harbin Institute of Technology (40) from China are the most contributive institutions. China and USA played central roles in cross-national cooperation, but the top three Chinese institutions displayed limited vitality in overseas communication. Rsc Advances (108) was the most productive journal followed by Desalination (97) and Desalination and Water Treatment (96). The research direction of NNs in wastewater treatment was bound up with new NNs. Novel preparation methods and nanostructures were powerful impetus for its progress. Nanomaterials like graphene, nanotube, magnetic nanoparticle, and silver nanoparticle were hotpots in this field. Current and potential application of NNs in wastewater treatment as well as challenges were reviewed based on bibliometric results. This study also provided researchers future-minded advice about research topic selection.

**Electronic supplementary material:**

The online version of this article (10.1186/s11671-018-2649-4) contains supplementary material, which is available to authorized users.

## Background

Since “nanomaterial” and “nanotechnology” were put forward, they have been the focuses of scientific field, both within and across disciplines. Arguably due to the continual research funding and scientific breakthroughs for nanometer domain, new NNs promote the development of areas such as chemistry [1] and materials science, medicine and pharmacology [2], electronics and photonics, environment and energy [3]. Moreover, NNs also play a vital role in contributing to wastewater treatment because of their high surface area and high reactivity [4, 5].

With the ever-increasing population improvement of living standard worldwide, massive effluent will pose serious challenges and burdens to our society [6]. Wastewater treatment system is the junction point between sewage and the natural sources of water such as rivers, lakes, reservoirs, and groundwater. Consequently, effectiveness of wastewater treatment system will produce a great impact on water recycling. In many cases, appropriate treatment of wastewater guarantees the safety of drinking water [7] and resources recovery [8]. Therefore, it is not exaggerative to make technological innovation a leading goal in wastewater treatment. Thankfully, NNs give us more options. The next-generation wastewater treatment and water supply system relying on NNs can be high-efficient [9], environmental-friendly, coproduct-free [10] as well as economically feasible [11]. NNs provide high performance in wastewater treatment with main application of adsorption [12], membranes and membrane process [13], photocatalyst, disinfection and microbial control, sensing and monitoring [14]. Considering the fact that increasing commercially engineered nanoparticles will find a final way into wastewater treatment plants, some researchers have shown concern about the possible influence on the disposal process [15]. For a better understanding of what role NNs are playing in the wastewater treatment, a quantitative and qualitative assessment is required for scientific guidelines.

In recent years, bibliometric method has been identified as a new strategy for figuring out useful points quickly and exactly from massive information. It can be used to evaluate the development of a domain mathematically during a given period. Zyoud et al. [16] offered guidance to future research on lithium toxicity by studying relative publications for nearly a century with bibliometric method. Zhang et al. [17] made a bibliometric analysis in water footprint and found that factors such as water-food-energy nexus and driving mechanism of water footprint variation promoted the development of this field. Yataganbaba et al. [18] introduced bibliometric measures into phase change material and encapsulation subjects and provided insights for the future research. CiteSpace is designed as a knowledge domain visualization software [19]. And the central concepts of this tool are burst detection, betweenness centrality, and heterogeneous networks [20]. In addition, it can present results in an easy understanding visual format through the diagrams [21]. As a result of above reasons, CiteSpace is gaining increasingly prevalence among scientific research workers [22, 23].

Given the exponential growth of significance and publication numbers, a critical analysis of its past, current, and future study is urgent. This paper attempts to investigate the development of NNs in wastewater treatment related scientific studies from 1997 to 2016 with combined technique of bibliometric and CiteSpace method.

## Methods

### Data Sources

The Web of Science Core Collection covers most of the important journals and widely applied in a variety of scientific fields [24, 25]. To obtain qualified information on topic of NNs in wastewater treatment, our data source was retrieved from Science Citation Index Expanded (SCI) database. Aiming at reliable and accurate records, “nano*” and “sewage treatment” or “wastewater treatment” or “sewage disposal” or “wastewater disposal” were used as searching strategy. The search was conducted on 30 June 2017, and publications were selected within the time span from 1997 to 2016. Then 2604 records were collected.

### Bibliometric Analysis

Bibliometrics is a comprehensive technique related to mathematical and statistical methods to figure out publication distribution, variation, and relationships quantitatively based on public databases [26]. With valid information divided, further analysis of literature characteristics and underlying knowledge will be possible.

Social network analysis is a useful tool for the representation and analysis of relational data [27]. It provides a quantitative measurement method on multiple relationships between different social roles. Geph is a prevalence software for social network analysis [28]. And in this study, it will be applied to display the cooperation networks among the top productive countries/territories and institutes.

### Visualization Analysis

Visualization analysis refers to presenting a large amount of data on a map by various network modeling tools [23]. In the following study, CiteSpace will be used for co-occurring keywords study. And ArcGIS will be employed to illustrate the distribution of the institutions worldwide.

## Results and Discussion

### The Characteristics of Research Publications

Of 2604 records about NNs in wastewater treatment, “Article” accounted for 91.90% (2393 records) while Review and Proceedings Paper contributed about 7.45% (194 records) and 5.45% (142 records), respectively. The records of other types made up less than 1% including meeting abstract, book chapter, news item, editorial material and correction. In this paper, only article is further studied.

The articles printing in English was 98.96% of total records, then Chinese 0.71%. The proportion of all other five languages including French, German, Malay, Polish, and Spanish was less than 0.4%. Considering many Chinese authors participated in the research of NNs in wastewater treatment, both English and Chinese papers are considered.

**Fig. 1:**
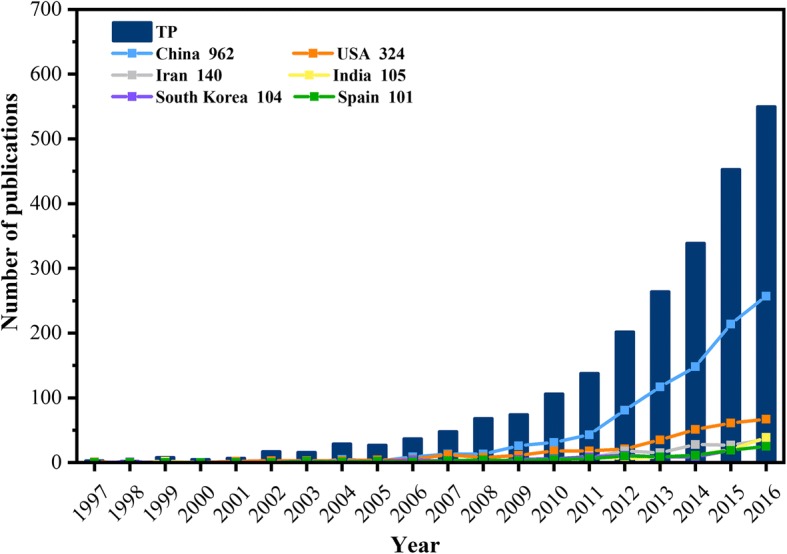
The annual publication number of top six productive countries during 1997–2016. TP: the total number of publications. The number after the country is the total publications of this country in this field over the time span

As observed in Fig. 1, the histogram shows the variations of articles related to NNs in wastewater treatment between 1997 and 2016. The first 5-year period witnessed a low level of publication number, with an average of 5 per year. Between 2002 and 2006, the mean publication number was about 25, just five-fold of last period. After a steady increase from 48 in 2007 to 74 in 2009, the annual publication record broke through 100 and reached 138 in 2011. During the next 5 years, the pace of publication rose rapidly and substantially. Therefore, it reveals that this topic has attracted an increasing interest in the scientific field. The fitting curves in Fig. 1 gave an idea of exponential growth in this domain. And the exact relationship between year (x) and number of publication (y) has been listed with a mathematic form. It warrants the research on NNs in wastewater treatment will remain a hot topic in coming years.

The line chart in Fig. 1 shows the annual publication output performance of the six most productive countries. Though at a low publishing level, the USA played a role of pioneer in this area over the first decade generally. After that, the publication number in China experienced and kept a robust rise in the following decade and took the leading position with 962 articles. This is owing to National Outline for Medium and Long-term Science and Technology Development Plan (2006–2020) that have taken environmental nanomaterial and nanotechnology to a strategic location. The growth trend was also observed in the USA, though with a much milder form. By the end of 2016, the USA had a total publication of 324. However, the number of papers on NNs in wastewater treatment by Iran (140), India (105), South Korea (104), and Spain (101) did not display clear increase until recent 5 years. And distinct gap could be observed between them with the two top countries. As a result, it could be illustrated that the highly productive countries contributed the overall development of this domain.

### The Contribution of Countries/Territories

Address and affiliations attached to every record can be deemed as effective information for countries/territories and institutes evaluation. Since authors’ addresses are missing, only 2391 articles were applied to analysis in this section. Over the two decades, 83 countries/territories had publication records on NNSs in wastewater treatment. And the top 20 countries/territories accounted for 83.95% of total publications.

In Table 1, the 20 most productive countries/territories were ranked according to their publication records, the number and percentage of publications without and within international collaboration, the number of publications published by first author and corresponding author, and h-index information. In every aspect, China showed an advantage over USA, the second productive country among the list. It was notable that USA got a considerable collaboration and h-index performance, with merely one-third publication number of China. Considering that h-index could be used as an indicator to measure both the impact and quantity of publication record, it indicated that USA possibly had a larger proportion of high-quality publications than China. Compared with USA, Iran fell behind distinctly in all aspects. And when it came to the rank of collaboration and h-index, Iran just took the twentieth and eleventh place respectively. Apart from China and USA, Australia, with a seventh place in total publication number, presented most activities than any other countries. Though showing no superiority in total publication number, Australia, Singapore, Germany, and Canada got relatively higher h-index ranks.

**Table 1:** Top 20 productive countries/territories during 1997–2016

Country	TP	TP R(%)	SP R(%)	CP R(%)	FP R(%)	RP R(%)	R(h-index)
China	962	1(40.22)	1(40.95)	1(37.64)	1(38.48)	1(38.45)	1(62)
USA	324	2(13.55)	2(9.08)	2(29.73)	2(9.12)	2(9.75)	2(55)
Iran	140	3(5.85)	3(6.73)	20(2.7)	3(5.56)	3(5.52)	11(20)
India	105	4(4.39)	4(4.11)	12(5.41)	4(3.93)	4(3.89)	7(23)
South Korea	104	5(4.35)	6(3.2)	4(8.49)	6(3.22)	6(3.31)	9(22)
Spain	101	6(4.22)	5(3.26)	6(7.72)	5(3.51)	5(3.47)	5(25)
Australia	95	7(3.97)	8(2.03)	3(11)	8(2.43)	8(2.47)	3(28)
Germany	84	8(3.51)	7(2.56)	10(6.95)	7(2.63)	7(2.55)	5(25)
Singapore	66	9(2.76)	11(1.55)	8(7.14)	10(1.84)	9(1.97)	3(28)
France	63	10(2.63)	19(1.17)	5(7.92)	17(1.38)	14(1.55)	13(17)
UK	63	10(2.63)	14(1.33)	7(7.34)	12(1.63)	12(1.67)	10(21)
Canada	62	12(2.59)	9(1.82)	12(5.41)	11(1.76)	10(1.88)	7(23)
Italy	53	13(2.22)	15(1.28)	11(5.6)	12(1.63)	13(1.63)	13(17)
Malaysia	51	14(2.13)	12(1.49)	15(4.44)	9(1.88)	10(1.88)	18(14)
Saudi Arabia	48	15(2.01)	26(0.59)	8(7.14)	25(0.75)	24(0.75)	16(15)
Japan	48	15(2.01)	15(1.28)	14(4.63)	14(1.46)	16(1.42)	13(17)
Taiwan	43	17(1.8)	10(1.71)	23(2.12)	14(1.46)	15(1.51)	16(15)
Switzerland	41	18(1.71)	15(1.28)	19(3.28)	19(1.3)	18(1.26)	12(18)
Turkey	41	18(1.71)	12(1.49)	22(2.51)	16(1.42)	16(1.42)	20(10)
Brazil	37	20(1.55)	18(1.23)	20(2.7)	18(1.34)	19(1.21)	19(11)

*TP*, the number of total publications; *SP*, the number of single country publications; *CP*, the number of internationally collaborative publications; *FP*, the number of publications as first author’s country; *RP*, the number of publications as corresponding author’s country; *R (%)*, the rank (the ratio of the number) of one country’s publications to the total number of publications for a certain aspect during 1997–2016. *R(h-index)*, the rank (the value of h-index) of a certain country’s publications during 1997–2016. The TP column was the numbers of publications. The *TP R(%)*, *SP R(%)*, *CP R(%)*, *FP R(%)*, *RP R(%)*, and *R(h-index)* columns provided information in the form of R (%) and R (%) as mentioned above

**Fig. 2:**
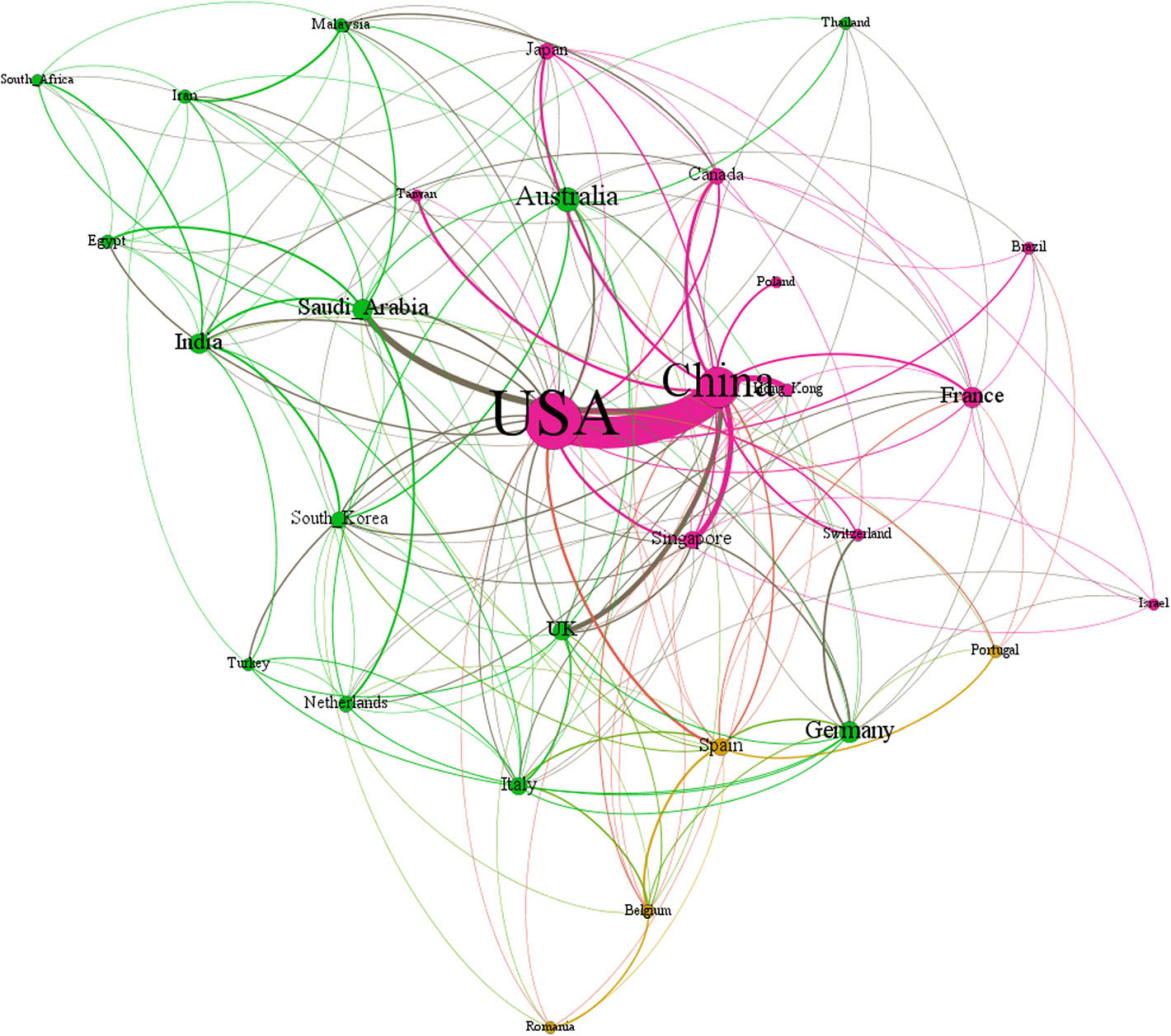
The cooperation network of the top 30 productive countries/territories

The social network analysis was then applied to analyze the coauthoring relationships among the top 30 productive countries/territories. And the results are displayed in Fig. 2. Notably, USA and China worked most closely among all countries/territories. They have produced 66 co-authored publications. In addition, the cooperation between China and Hong Kong, Saudi Arabia, and the UK were also remarkable. Unlike China which made relatively intense cooperation with certain countries/territories, USA kept connection with a larger range of countries/territories though with less density.

### The Contribution and Distribution of Institutes

According to information of author addresses, 1871 institutions have been showing interest in NNs in wastewater treatment. And Additional file 1: Figure S1 illustrated all retrieved institutions across the world. Areas with high distribution density mainly came from three main economic regions, namely, Europe, East Asia, and North America. Europe had the largest number of institutions followed by East Asia and North America respectively.

As listed in Table 2, about two-thirds of the top 30 institutes were from China, and two from Singapore, one from Iran, Malaysia, and USA. Chinese Academy of Sciences contributed the most publications in this area (149) followed by Tongji University (49) and Harbin Institute of Technology (40). More than half of total articles (54.75%) involved multi-institutional collaborations. The rank of publication number as to first author’s institute, corresponding, and author’s institute were in accordance with the total publication number generally. While it was noteworthy that Zhejiang University and Arizona State University, not performing remarkably in total publication number, ranked the eighth and ninth place in that of first author’s country and corresponding author’s country, respectively. In terms of h-index rank, Chinese Academy of Sciences kept the first place. Nevertheless, National University of Singapore, Tonji University, Shanghai Jiao Tong University, and Nanyang Technological University with various rank had similar h-index (16 or 17).

**Table 2:** The top 20 productive institutes during 1997–2016

Institution name	TP	TP R(%)	SP R(%)	CP R(%)	FP R(%)	RP R(%)	R(h-index)
Chinese Acad Sci, China	171	1(7.15)	2(2.13)	1(11.31)	1(3.01)	1(3.01)	1(30)
Tongji Univ, China	49	2(2.05)	1(2.4)	4(1.76)	2(1.76)	2(1.67)	3(16)
Harbin Inst Technol, China	40	3(1.67)	11(0.92)	2(2.29)	3(1.13)	3(1.17)	8(14)
Islamic Azad Univ, Iran	38	4(1.59)	6(1.2)	3(1.91)	6(1)	6(1)	13(11)
Natl Univ Singapore, Singapore	37	5(1.55)	4(1.39)	5(1.68)	4(1.05)	3(1.17)	2(17)
Shanghai Jiao Tong Univ, China	32	6(1.34)	5(1.29)	9(1.38)	4(1.05)	5(1.09)	3(16)
Tsinghua Univ, China	31	7(1.3)	9(1.02)	6(1.53)	8(0.92)	8(0.88)	9(12)
Dalian Univ Technol, China	27	8(1.13)	3(1.57)	25(0.76)	6(1)	7(0.96)	13(11)
Univ Sci and Technol China, China	27	8(1.13)	15(0.74)	7(1.45)	13(0.71)	12(0.71)	6(15)
Nanjing Univ, China	27	8(1.13)	15(0.74)	7(1.45)	10(0.79)	9(0.79)	9(12)
Zhejiang Univ, China	25	11(1.05)	11(0.92)	10(1.15)	8(0.92)	11(0.75)	9(12)
Jiangsu Univ, China	22	12(0.92)	8(1.11)	25(0.76)	10(0.79)	15(0.67)	19(8)
Nanyang Technol Univ, Singapore	22	12(0.92)	21(0.65)	10(1.15)	16(0.63)	17(0.63)	3(16)
Univ Teknol Malaysia, Malaysia	22	12(0.92)	15(0.74)	13(1.07)	16(0.63)	12(0.71)	18(9)
Hunan Univ, China	21	15(0.88)	21(0.65)	13(1.07)	19(0.59)	20(0.54)	9(12)
Arizona State Univ, USA	21	15(0.88)	6(1.2)	45(0.61)	10(0.79)	9(0.79)	6(15)
Shandong Univ, China	20	17(0.84)	11(0.92)	25(0.76)	16(0.63)	17(0.63)	15(10)
Tianjin Univ, China	20	17(0.84)	21(0.65)	16(0.99)	15(0.67)	15(0.67)	19(8)
Huazhong Univ Sci and Technol, China	19	19(0.79)	21(0.65)	20(0.92)	19(0.59)	19(0.59)	15(10)
Jilin Univ, China	19	19(0.79)	9(1.02)	45(0.61)	13(0.71)	12(0.71)	15(10)

*TP*, the number of total publications; *SP*, the number of single institute publications; *CP*, the number of internationally collaborative publications; *FP*, the number of publications as first author’s institute; *RP*, the number of publications as corresponding author’s institute; *R (%)*, the rank (the ratio of the number) of one country’s publications to the total number of publications for a certain aspect during 1997–2016. *R(h-index)*, the rank (the value of h-index) of a certain institute ‘s publications during 1997–2016. The *TP* column was the number of publications. The *TP R(%)*, *SP R(%)*, *CP R(%)*, *FP R(%)*, *RP R(%)*, and *R(h-index)* columns provided information in the form of R (%) and R (%) as mentioned above

**Fig. 3:**
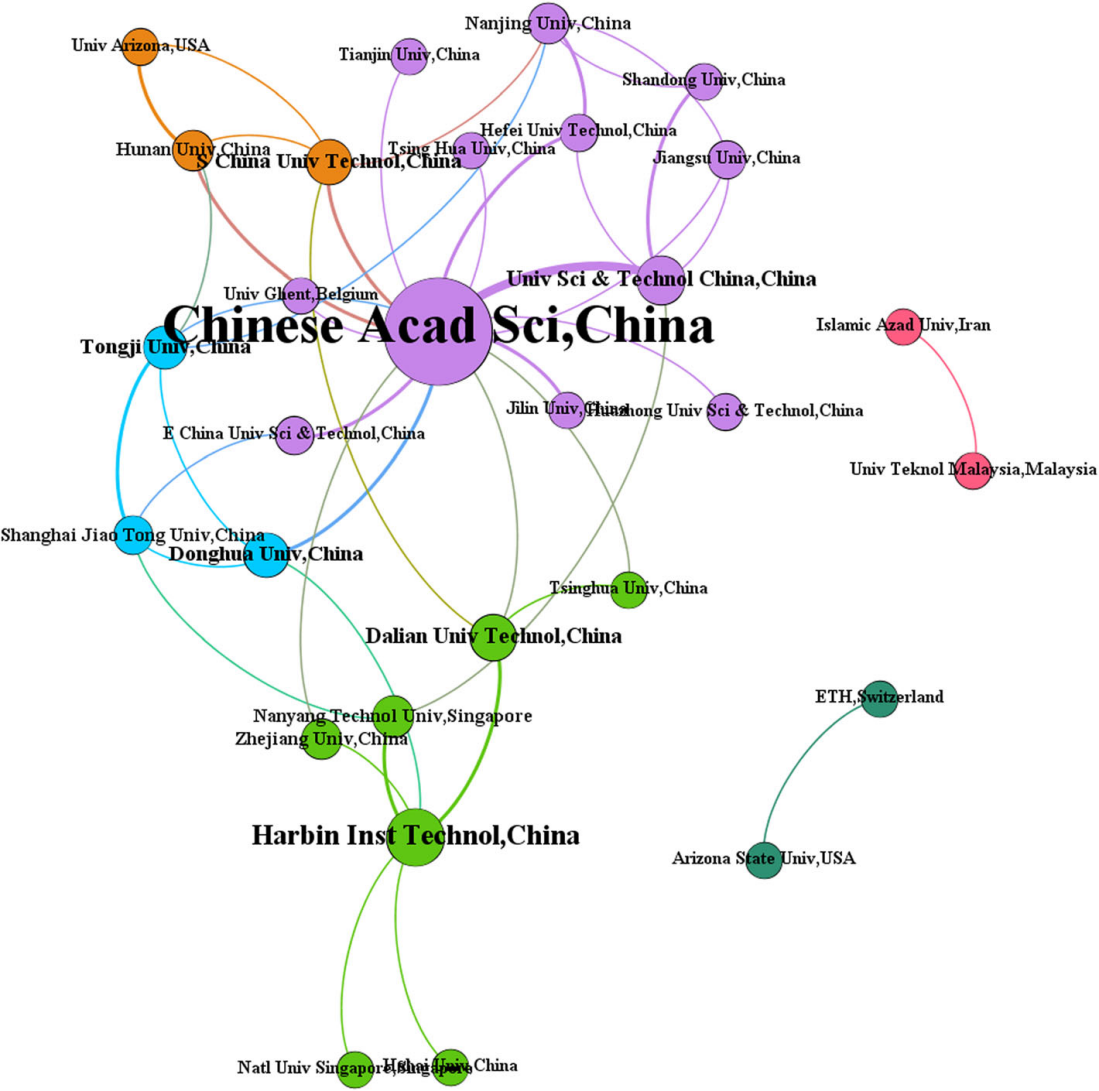
The cooperation network of the top 30 productive institutions

As shown in Additional file 1: Figure S2, Chinese Academy of Sciences, China and University of Science and Technology of China had a strong cooperation relationship. It was worth noting that a large proportion of collaboration was among Chinese institutions. As the center of the network, Chinese Academy of Science had partnerships with almost all domestic institutions but limited communication with the overseas. Besides that, Harbin Institute of Technology, China, also cooperated well with other six institutes. In addition, ETH, Switzerland and Arizona State University, Islamic Azad University and University Teknol Malaysia showed collaboration with each other only but lost connection with the whole cooperation network. It was necessary to point out that Duke University from USA was not found in Fig. 3. This meant it did not cooperate with the rest of top 30 institutions.

The annually publication of the top five productive institutes over the two decades illustrated in Additional file 1: Figure S2. Before 2005, almost no publishing record was found among these top five institutes. Since then, the publication number grew rapidly despite obvious fluctuation in some years. It was in the year of 2011 that Chinese Academy of Sciences took a giant step forward and surpassed other four institutes. After that, it kept a higher growth rate and ranked on top during the last 5 years. The trend among these five most productive institutes suggested that this field has increasingly become the focus of researchers’ attention worldwide.

### The Distribution of Subject Categories and Journals

All retrieved articles are divided up among 44 subject categories. As listed in Table 3, engineering ranked the first with 1069 records followed by chemistry with 757 records and environmental sciences and ecology with 702 records respectively. As illustrated in Additional file 1: Figure S3, the number of top six productive subject categories climbed steadily after barely growing over the first 5-year period. Before 2015, engineering rose at a relatively high pace and took a leading place continuously. However, chemistry showed a staggering growth after 2011 and took an advantage over environmental sciences and ecology, and engineering in 2014 and 2016, respectively. The possible reason was that researchers have realized the significance of chemical mechanism of NNs’ behavior in wastewater treatment. The consistent increase in environmental sciences and ecology, and water resources implied the important and potential effect of NNs in environmental area. The prosperity of engineering, materials science, and science and technology—other topics maybe due to the emerging NNs.

**Table 3:** The 15 most productive subjects during 1997–2016

Subject	TP	Percent
Engineering	1069	44.67
Chemistry	757	31.63
Environmental sciences and ecology	702	29.34
Materials science	425	17.76
Water resources	416	17.38
Science and technology—other topics	236	9.86
Physics	179	7.48
Polymer science	133	5.56
Energy and fuels	87	3.64
Biotechnology and applied microbiology	79	3.3
Biochemistry and molecular biology	49	2.05
Electrochemistry	49	2.05
Marine and freshwater biology	39	1.63
Agriculture	30	1.25
Toxicology	26	1.09

*TP*, the number of total publications

The 2393 articles are divided into 449 journals. And as listed in Table 4, the contribution of top 20 journals to all publications was 47.20%. Rsc Advances, a comprehensive journal for chemical sciences, was the most productive journal with 108 records followed by Desalination, and Desalination and Water Treatment with 97 and 96 respectively. Enjoying a very high reputation in environment domain worldwide, Environmental Science and Technology ranked seventh with 76 records. It implied that NNs is increasingly concerned as environmental issues. Additionally, it was obvious that most journals listed in Table 4 had a high Impact factor (IF) value, with 65% of that ranging from 4.2 to 9.5. In general, IF is considered as an effective indicator for the quality of a journal [29]. Therefore, it suggested the prevalence of this topic among outstanding scientists.

**Table 4:** The 20 most productive journals during 1997–2016

Journal Name	R	TP	Percent	IF 2016
Rsc Advances	1	108	4.52	3.108
Desalination	2	97	4.06	5.527
Desalination and Water Treatment	3	96	4.01	1.631
Chemical Engineering Journal	4	87	3.64	6.216
Water Research	5	79	3.3	6.942
Journal of Hazardous Materials	6	78	3.26	6.065
Environmental Science and Technology	7	76	3.18	6.198
Journal of Membrane Science	8	72	3.01	6.035
Water Science and Technology	9	70	2.93	1.197
Separation and Purification Technology	10	59	2.47	3.359
Chemosphere	11	38	1.59	4.208
Environmental Science and Pollution Research	11	38	1.59	2.741
Applied Surface Science	13	36	1.51	3.387
Acs Applied Materials and Interfaces	14	32	1.34	7.504
Science of the Total Environment	15	31	1.3	4.9
Applied Catalysis B-Environmental	16	28	1.17	9.446
Industrial and Engineering Chemistry Research	17	27	1.13	2.843
Journal of Materials Chemistry A	18	26	1.09	8.867
Journal of Colloid and Interface Science	19	25	1.05	4.233
Bioresource Technology	19	25	1.05	5.651

*TP*, the number of total publications

Additional file 1 Figure S4 showed the publication performance of the top five journals. Clearly, the last 2 and 3 years witnessed the soaring trends of Rsc Advances, and Desalination and Water treatment. Desalination, however, suffered a sharp fall in 2012 and kept an even lower level afterward than that in period from 2005 to 2010. Moreover, the other four journals increased in fluctuation over the whole time span. It demonstrated that the research of NNs in wastewater treatment has been developing into a cross-over study widely.

### The Main Research Fields

Keywords of a paper can offer effective points related to its main ideas. Burst detection in CiteSpace can retrieve bursts keywords as signs of emerging trends of NNs in wastewater treatment [30]. In this section, only 2386 records were analyzed, because another 7 records were invalid with incomplete information.

**Fig. 4:**
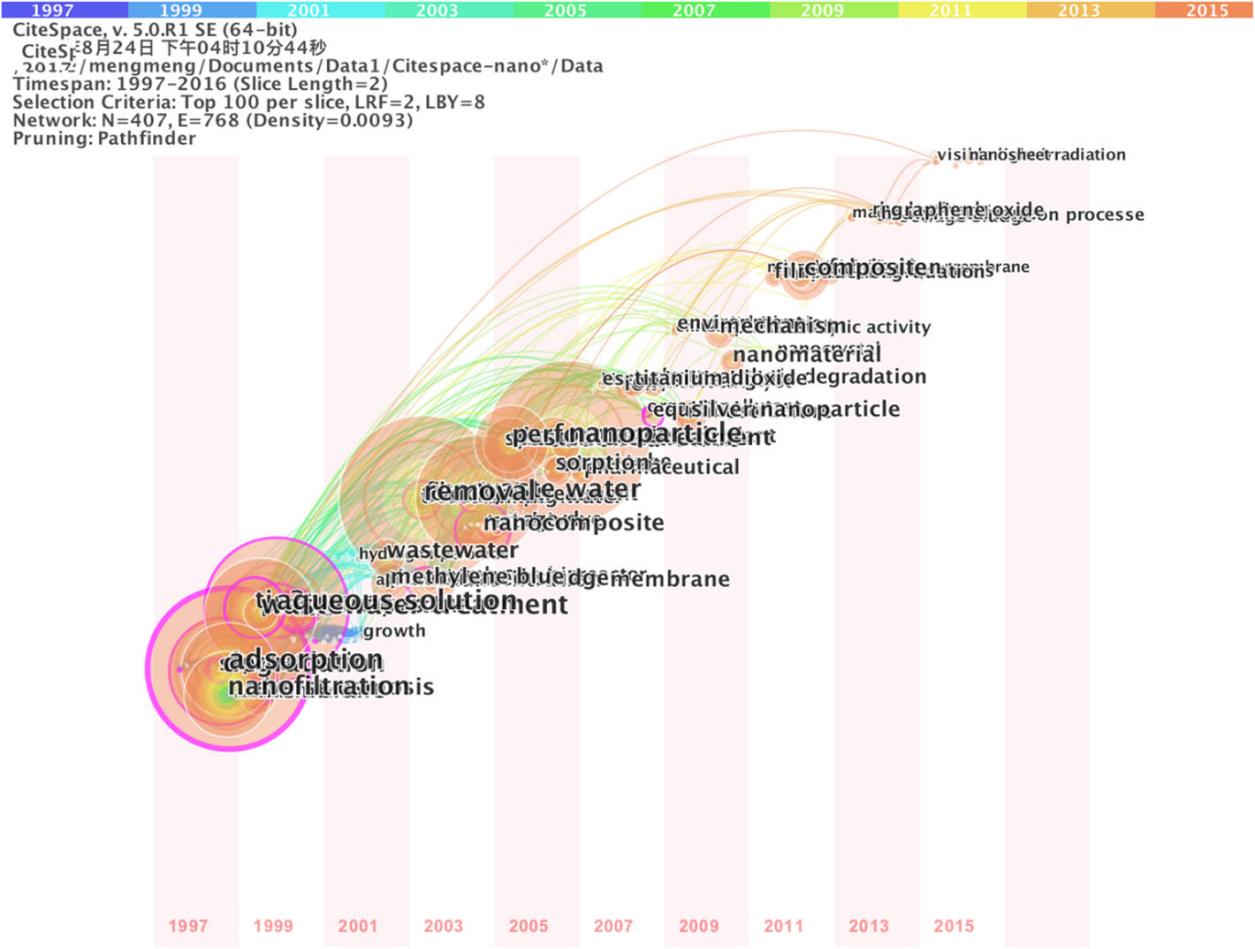
Timeline view of a network related to co-occurring keywords

A timeline visualized network based on keywords was shown in Fig. 4. The color of both circle and line in the network were corresponding to consecutive years on the top of the figure itself. Each dot represented a node in the network. And the nodes are keywords. Lines between nodes suggest co-occurring links. It needs to be emphasized that highlight nodes with significant research fields are marked out with purple trims. And a thicker purple trims suggest a higher frequency of co-occurrence [31]. Notably, *adsorption* (430), *degradation* (306), *nanofiltration* (264), *reverse osmosis* (132), *membrane* (130), *TiO*_*2*_ (183), *photocatalysis* (124), *ultrafiltration* (114), r*emoval* (461) and *nanocomposite* (157) were high-frequency keywords appearing in an earlier stage.hile, *carbon nanotube* (120), *sorption* (96), TiO_2_ *nanotube* (72), *photocatalytic degradation* (71), *photocatalyst* (55), *silver nanoparticle* (103), *composite* (139), *hydrothermal synthesis* (34), *graphene oxide* (60), *graphene* (43), *sewage sludge* (37), *transformation* (34) and *magnetic nanoparticle* (33) were frequently used keywords lately. It demonstrated that they were research focuses of NNs in wastewater treatment.

Keywords burst are an effective indicator of research hotspot in the discussed area [22]. The keywords burst suggest a particular keyword is in contact with an increasing number of other keywords and has obtained great attention from the scientific field. Table 5 listed the top 20 keywords with the strongest bursts during 1997 to 2016. The year in the last column represented the certain period of a keywords burst. A large number of keywords started to spring up since 1998. And top three strongest were *nanofiltration*, r*everse osmosis*, and *ultrafiltration* with long bursting-out period of 14, 10 and 13 years respectively. Taking *nanofiltration*, for example, the burst began in 1998 and ended in 2011. It meant *nanofiltration* had received particular attentions and used to be a research hotspot in the course from 1998 to 2011. Generally, the keywords bursts period showed accordance with the results in Fig. 4. Similarly with high-frequency keywords, most of the keywords bursts were about the core nanotechnology of water purification and the application of NNs. Besides, the most recent bursts of keyword were *composite*, *graphene*, and *sewage sludge*. It might suggest that composite nanomaterials and graphene are emerging trend. Meanwhile, the scope of the NNs studies is extending to sewage sludge research.

**Table 5:** listed the top 20 keywords with the strongest bursts

NO	Keyword	Burst	Time span
1	Nanofiltration	26.46	1998–2011
2	Reverse osmosis	18.92	2002–2010
3	Ultrafiltration	14.16	1999–2011
4	Aquatic environment	11.66	2002–2012
5	Thin film	11.56	2013–2014
6	Rejection	10.32	2005–2010
7	Microfiltration	10.21	1990–2012
8	Composite	8.69	2014–2016
9	Behavior	8.64	2002–2012
10	Ultrafiltration membrane	8.57	2002–2012
11	Fouling	8.49	2000–2012
12	Pretreatment	7.76	2007–2012
13	Exposure	7.73	2009–2012
14	Silver	6.66	2013–2014
15	Graphene	6.32	2014–2016
16	Filtration	6.28	2003–2012
17	Sewage sludge	6.26	2014–2016
18	Dissolution	6.21	2013–2014
19	Morphology	6.11	2011–2014
20	Product	6.11	2011–2014

### The Most Highly Cited Articles

Most highly cited publications are also useful index to demonstrate the research interest and hotpot of a scientific field [32]. The 10 most highly cited publications during the time span, as well as 3 top ones in every 3 years nearly were all listed in Table 6. Environmental science and technology, and water research were the most prevalent journals for the top 10 highly cited articles, with 5 and 3 on each respectively. USA contributed most to all listed citations, and China came to be the second.

**Table 6:** Most highly cited articles during 1997–2015

PY	TC-2016	TC/Y	TI	SO	CC1
2009	1001	125	Modeled environmental concentrations of engineered nanomaterials (TiO2, ZnO, Ag, CNT, Fullerenes) for different regions [57]	Environmental Science and Technology	Switzerland
2008	884	98	Nanoparticle silver released into water from commercially available sock fabrics [58]	Environmental Science and Technology	USA
2007	615	62	Occurrence and removal of pharmaceuticals and endocrine disruptors in South Korean surface, drinking, and waste waters [59]	Water Research	USA; South Korea
2008	516	57	The inhibitory effects of silver nanoparticles, silver ions, and silver chloride colloids on microbial growth [60]	Water Research	USA
2012	502	100	Titanium dioxide nanoparticles in food and personal care products [61]	Environmental Science and Technology	USA; Switzerland; Norway
2001	481	30	Study of Au/Au3 + -TiO2 photocatalysts toward visible photooxidation for water and wastewater treatment [62]	Environmental Science and Technology	Hong Kong
2008	462	51	Estimation of cumulative aquatic exposure and risk due to silver: contribution of nano-functionalized plastics and textiles [63]	Science of the Total Environment	Switzerland
2001	385	24	Estrogenic potency of chemicals detected in sewage treatment plant effluents as determined by in vivo assays with Japanese medaka (*Oryzias latipes*) [64]	Environmental Toxicology and Chemistry	Canada; USA
2009	377	47	Titanium nanomaterial removal and release from wastewater treatment plants [65]	Environmental Science and Technology	USA
2005	355	30	Adsorption thermodynamic, kinetic, and desorption studies of Pb2+ on carbon nanotubes [12]	Water Research	UK; China
2016	41	41	Highly efficient simultaneous ultrasonic-assisted adsorption of brilliant green and eosin B onto ZnS nanoparticles loaded activated carbon: artificial neural network modeling and central composite design optimization [66]	Spectrochimica Acta Part A-Molecular and Biomolecular Spectroscopy	Iran
2016	35	35	Magnetic magnetite (Fe3O4) nanoparticle synthesis and applications for lead (Pb2+) and chromium (Cr6+) removal from water [67]	Journal of Colloid and Interface Science	India; USA
2016	35	35	Graphene-based microbots for toxic heavy metal removal and recovery from water [68]	Nano Letters	Germany; Spain; Singapore
2015	107	54	Polymer-matrix nanocomposite membranes for water treatment [69]	Journal of Membrane Science	USA
2015	44	22	Preparation of graphene oxide-based hydrogels as efficient dye adsorbents for wastewater treatment [70]	Nanoscale Research Letters	China
2015	41	21	Graphene oxides for simultaneous highly efficient removal of trace level radionuclides from aqueous solutions [71]	Science China-Chemistry	China; Saudi Arabia
2014	94	31	Adsorptive removal of methylene blue by rhamnolipid-functionalized graphene oxide from wastewater [72]	Water Research	China; USA
2014	92	31	Aqueous adsorption and removal of organic contaminants by carbon nanotubes [73]	Science of the Total Environment	China; Canada
2014	92	31	Fate of zinc oxide and silver nanoparticles in a pilot wastewater treatment plant and in processed biosolids [74]	Environmental Science & Technology	USA; France; UK

*TC*, total citations; *TC/Y*, average annual citations since publication

By analyzing the citations, it was found that the usage of various NNs for contaminants removal form wastewater has consistently kept as a hot field. New nanomaterial development and application were popular subjects among all mentioned articles. In general, the hotpots found according highly cited publications showed similar trend as that in part 3.5. And this was especially obvious in terms of highly cited papers in recent 3 years. Of the nine papers, four studies were based on *graphene* utilization in wastewater treatment. In addition, *magnetic nanoparticle*, *carbon nanotubes*, and s*ilver nanoparticles* were also listed in the left five papers. Besides, it should be note that the negative effects of nanomaterials to both human beings and the environment have also attracted researchers’ concern. It suggested researchers have been considering nanomaterials in a rational way, though the evolution it has been bringing to our society.

### Current and Potential Application of NNs in Wastewater Treatment

Adsorption, membrane filtration, and sensing and detection were four focuses in 3.4–3.6 based on bibliometric analysis. It was based on main functions of NNs in wastewater treatment. Though increasing and emerging water contamination from multitudinous sources, the mechanism we turned to for problems elimination varied little. Thus, we critically reviewed the present and future of NNs from four categories mentioned above. The potential risk of NNs was not be elaborated here for transcending the application realm.

#### Adsorption

Adsorption was a preferred choice over other water strategies for its simplicity in operation and the universality for common organic and inorganic contaminants [33]. Size-dependent nanostructures guaranteed nanomaterials inherent advantages in comparable specific surface area or active sites, which were longstanding bottlenecks for conventional adsorbents. Carbon-based nano-adsorbents, typically carbon aerogels [34], carbon nanotubes (CNTs) [35], graphene [36], and their hybridization states [37] were promising for wastewater treatment, and their excellent performance for heavy metals and organic contaminants removal have demonstrated generally. For hydrophobicity of their graphitic surface, carbon-based nano-adsorbents formed loose aggregates, which reduced the effective surface area and increased adsorption energy. Though functional groups or metal oxide nanoparticles were introduced to eliminate this drawback, their complete recovery from water after the adsorption process remained operational cost [38]. Regeneration and reuse of carbon-based nano-adsorbents could be achieved by reducing the aqueous pH [39]. The adsorption capacity was relatively stable after regeneration. Although impressive progress has taken place over the past years, the production and purification steps often introduced contaminants and impurities, and even cause structure degradation. Besides, the synthesis of carbon nanoparticles with a desired or homogeneous porous network is still a main challenge for all research in this field.

In the form of powder, nano-adsorbents are readily integrated into existing treatment processes in any slurry reactors relating to mixing process. While a matched separation technology is required to separate and recover the nano-adsorbents. Some improvements have been made to fix the nanoparticles in pellets/beads to form a nano-loaded system. The further separation process can be omitted under the circumstances, problems about mass transfer limitations and head loss, however, will arise.

#### Membrane Filtration

As a common constituent of water and wastewater treatment system, membrane processes was divided into microfiltration (MF), ultrafiltration (UF), and nanofiltration (NF) based on their size [40]. Since the key part of membrane processes was filtration material, NNs were contributing to more efficient water filtration processes (nanofiber membranes, nanocomposite membranes, thin film nanocomposite (TFN) membranes) [40]. The high energy consumption, lifetime reduction, and filtration failure lead by membrane fouling were major challenges of membrane processes. Modified membranes with functional nanomaterials were regarded as a promising opportunity to face this dilemma. By decorating with inorganic nanoparticles, such as alumina [41], silic [42], zeolite, and TiO [43], membrane hydrophilicity [44] was increased to avoid fouling. TFN membrane, a new concept form Hoek’s research group, was initiated by embedding zeolite NaA nanoparticles within polyamide layer to form composite membrane [45]. And a significant membrane flux enhance was achieved compared to the common TFC membrane [45]. However, meaningful study on how nanomaterials improve the characteristics of polyamide layer of TFN membrane was expected in this field. And nano-Ag was also added on polymeric membranes to prevent biofilm formation [46] and kill viruses [47] on the membrane surface. TiO_2_-based nanomaterials and metallic/bi-metallic catalyst nanoparticles such as nano zero-valent iron (nZVI) were common catalyst toward contaminant degradation, thus incorporating them into membranes would effectively relieve residue retention.

#### Sensor and Detection

A vast number of synthetic organic compounds, such as PAHs, PCB, and PBDEs, caused water pollution in an extremely low concentration. A major challenge for wastewater treatment was sensing and detecting them rapidly and accurately. For many, nanomaterials were excellent adsorbents; they concentrated pollution to meet the detection threshold. CNTs have been used in real water samples for organic compounds detection [48]. Au-TiO_2_ nanocomposite showed good linear with trace organophosphates (OPs) insecticides at 1.0 ng/ml level [49]. Multifunctional nanotube array based on TiO_2_ was used to detect herbicide 4-chlorophenol (4-CP), dichlorophenoxyacetic acid (2,4-D) and methyl-parathion (MP) [50]. Pathogen and virus have also been regarded as a long-term threat in wastewater. Due to large surface/volume ratios in nano-size devices, NNs depended nano-biosensors were fast and well-timed in some pathogen and virus diagnosis. Quantum dots [51], carbon nanotubes [52], graphene oxide [53], silica [54], and metal nanoparticles [55, 56] were solid foundation for sensor and detection technologies. The present challenges are aimed at eliminating false detection of pathogen and virus in complicated wastewater sample. In addition, research and development on portable and reusable detector will also be a creative endeavor.

## Conclusions

Bibliometric technique was applied to investigate the development of NNs in wastewater treatment. The number of publications experienced an exponential increase during the examined two decades. China was the most productive country and made up 40.22% (962) of total articles with the highest h-index (62). However, the USA, with merely one-third publication number of China, achieved an approximate h-index (55). In addition, both China and USA also took overwhelming advantages over other countries/territories in international collaboration. And strongest cooperation relationships were observed between them. Chinese Academy of Science had strongest collaborative ability, but it showed limited communication with overseas institutions.

Graphene, nanotube, magnetic nanoparticle, and silver nanoparticle are hotpots in recent years. And NNs is developing toward a more detailed and sophisticated classification in spatial structure. Different from traditional NNs, nanocomposites with multicomponent or multi-element emerged with optimization and precise control of processing. Researchers are trying to design nanomaterials rather than to prepare them. However, when it comes to practical application, wider commercialization of NNs is urgently needed. Nanoparticles with hazardous and toxic bring risk to environmental safety and public health. Synchronous recovery technology is urgently needed to eliminate its negative effects and realize resources recycle. Though some NNs have been widely applied in water and wastewater treatment, we are far from making the most of them commercially.

## Additional file

Additional file 1: **Figure S1.** The geographical distribution of institutions. **Figure S2.** The number of publications of the top five productive institutions during 1997–2016. TP: the total number of publications. **Figure S3.** The number of articles of the top six productive subject categories. **Figure S4.** The number of articles of the top five productive journals during 1998–2013. (DOCX 93742 kb)

## Abbreviations

CP: The number of internationally collaborative publications
FP: The number of publications as first author’s country
NNs: Nanomaterials and nanotechnologies
R (%): The rank (the ratio of the number) of a certain item
R(h-index): The rank (the value of h-index) of a certain item’s
RP: The number of publications as corresponding author’s country
SP: The number of single country publications
TP: The number of total publications
