# Supplementary material for: The Role of Nanomaterials and Nanotechnologies in Wastewater Treatment: a Bibliometric Analysis
Source: Nanoscale Res Lett. 2018 Aug 10;13:233. doi: 10.1186/s11671-018-2649-4 (PMC6086776; doi:10.1186/s11671-018-2649-4)
Supplement: Supplementary file 1 — Figure S1. The geographical distribution of institutions. Figure S2. The number of publications of the top five productive institutions during 1997–2016. TP: the total number of publications. Figure S3. The number of articles of the top six productive subject categories. Figure S4. The number of articles of the top five productive journals during 1998–2013. (DOCX 93742 kb) [file 11671_2018_2649_MOESM1_ESM.docx]

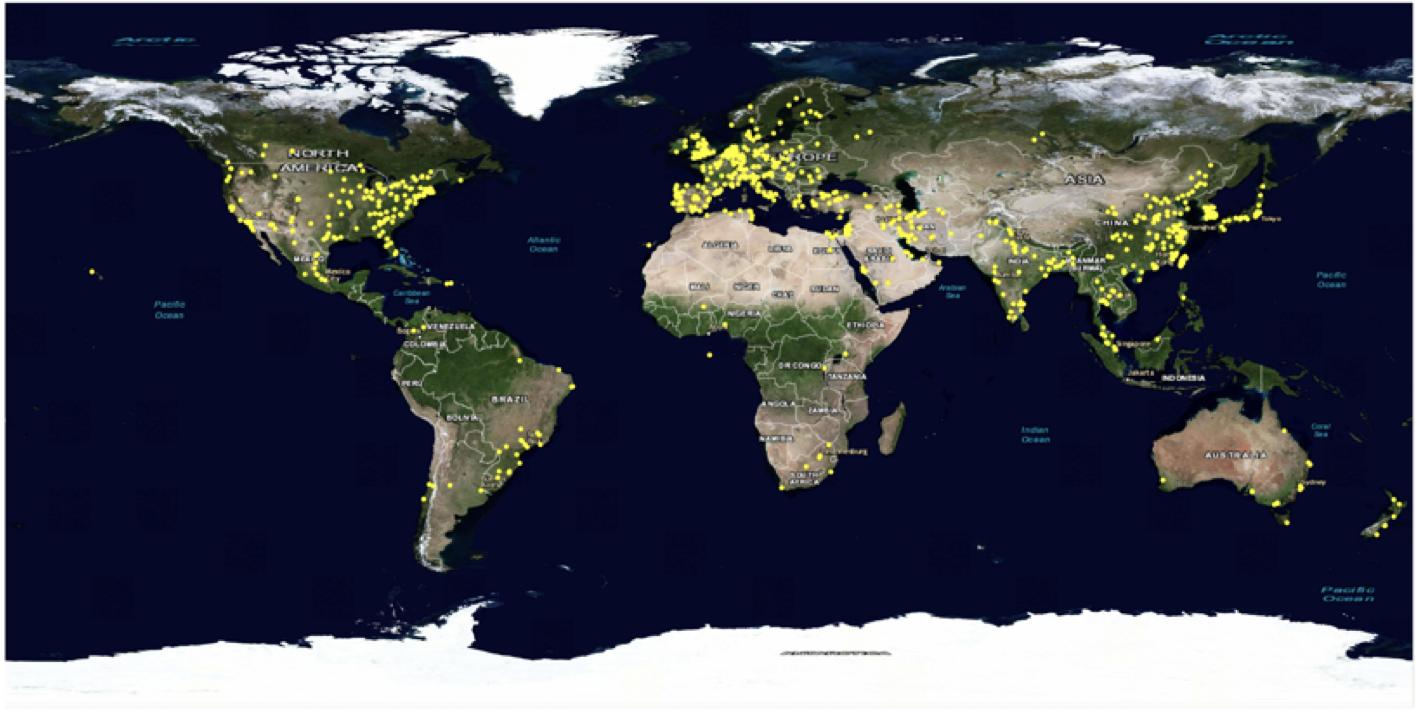


Figure S1 The geographical distribution of institutions.

Figure S2 The number of publications of the top 5 productive institutions during 1997–2016. TP: the total number of publications.

Figure S3 The number of articles of the top 6 productive subject categories.

Figure S4 The number of articles of the top 5 productive journals during 1998–2013.
